# Neurotoxicity of Chronic Alcohol Exposure: Mechanistic Insights, Cellular Disruption, and Emerging Therapeutic Strategies

**DOI:** 10.3390/ijms27010299

**Published:** 2025-12-27

**Authors:** Philip Gołaszewski, Agata Wawrzyniak, Maksymilian Kłosowicz, Aleksandra Burbelka, Krzysztof Balawender

**Affiliations:** 1 Department of Histology and Embryology, Faculty of Medicine, University of Rzeszów, 35-516 Rzeszów, Polandawawrzyniak@ur.edu.pl (A.W.); 2 Department of Normal and Clinical Anatomy, Faculty of Medicine, University of Rzeszów, 35-516 Rzeszów, Poland

**Keywords:** alcohol-induced neurotoxicity, ethanol metabolism, mitochondrial dysfunction, oxidative stress, neuroinflammation, epigenetics, apoptosis, PI3K/Akt/mTOR pathway

## Abstract

Chronic alcohol exposure induces complex pathophysiological changes in the central nervous system (CNS), contributing to the onset and progression of neurodegenerative disorders. This review integrates recent findings on ethanol-induced neurotoxicity, focusing on key mechanisms including oxidative stress, protein misfolding, mitochondrial dysfunction, calcium dysregulation, neuroinflammation, and epigenetic alterations. We further discuss the detrimental impact of ethanol metabolism particularly its neurotoxic intermediates such as acetaldehyde and salsolinol on neuronal integrity. Special emphasis is placed on glial cell activation, blood–brain barrier disruption, and dysregulation of survival pathways such as PI3K/Akt/mTOR. Finally, we highlight promising therapeutic strategies targeting these molecular and cellular disruptions. A comprehensive understanding of these processes is critical for the development of neuroprotective interventions aimed at mitigating alcohol-related cognitive and functional decline.

## 1. Introduction

Alcohol remains one of the most widely consumed psychoactive substances worldwide. While moderate intake may not lead to overt neurotoxicity, chronic and binge drinking are consistently associated with progressive structural and functional alterations within the central nervous system (CNS) [1,2]. Ethanol exerts its detrimental effects across multiple brain regions, including the prefrontal cortex, hippocampus, and cerebellum, leading to impairments in cognition, emotional regulation, executive function, memory, and motor coordination [2,3]. These effects are particularly pronounced in vulnerable populations, such as adolescents with immature neural circuitry, older adults with reduced neuroplasticity, and individuals with comorbid psychiatric or metabolic disorders [2,4]. The neurotoxic impact of ethanol arises from a multifaceted and interconnected network of pathophysiological processes. Key mechanisms include the generation of reactive oxygen and nitrogen species (ROS/RNS), disruption of mitochondrial integrity and energy production [5,6], protein misfolding and proteostasis failure [7], intracellular calcium imbalance [8], excitotoxicity [9], neuroinflammatory signalling [10,11], and epigenetic remodeling [12,13]. Ethanol metabolism, through enzymatic and non-enzymatic pathways, yields neurotoxic metabolites such as acetaldehyde and salsolinol, which further exacerbate oxidative stress and dopaminergic vulnerability [14,15,16]. Chronic alcohol exposure also compromises the blood–brain barrier [17], disrupts glial function [18], and promotes sustained neuroimmune activation [10,11]. Importantly, these changes may persist even after cessation of alcohol intake [13,19], highlighting the insidious and self-perpetuating nature of alcohol-induced neurodegeneration. Emerging evidence also points to the involvement of peripheral–central interactions, including the gut–brain and liver–brain axes, in shaping the neurotoxic landscape of alcohol use disorders (AUD) [3,20]. Consistent with these systemic vulnerabilities, recent work has highlighted that chronic alcohol consumption engages molecular pathways implicated in Alzheimer’s disease, Parkinson’s disease, and amyotrophic lateral sclerosis, underscoring its relevance as a modifiable contributor to major neurodegenerative disorders [21].

**Figure 1:**
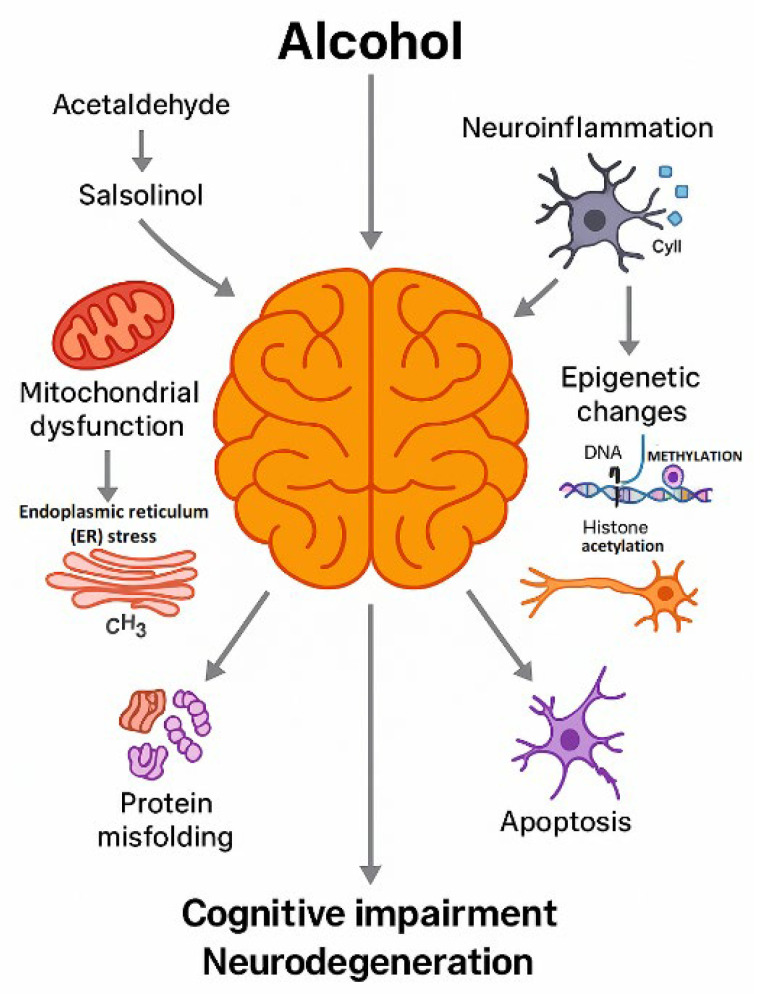
Conceptual framework of the review. This schematic provides an overview of how the review is organized. The diagram illustrates the progression from chronic ethanol exposure and the formation of toxic metabolites (acetaldehyde and salsolinol), through the resulting cellular and molecular disturbances (mitochondrial dysfunction, endoplasmic reticulum stress, protein misfolding, neuroinflammation, epigenetic changes and apoptosis), to structural and functional alterations in the brain (cognitive impairment and neurodegeneration). Arrows indicate the sequential relationships between these levels of organization and their convergence on neuronal and glial injury. The final part of the scheme points to neuroprotective and therapeutic strategies discussed in the later sections, so that the figure serves as a roadmap to the subsequent chapters of the review. Created by the authors in Adobe Illustrator version 27.5, 2023 (created on 27 October 2025).

Understanding the complex molecular and cellular sequelae of chronic alcohol exposure is essential for the identification of mechanistically informed therapeutic targets. Accordingly, this review synthesizes contemporary evidence on the major drivers of ethanol-induced neurotoxicity, including oxidative injury, mitochondrial dysfunction, protein misfolding, neuroinflammation, calcium dysregulation, apoptosis, and epigenetic alterations. We also highlight current and emerging therapeutic strategies aimed at mitigating neuronal damage and promoting functional recovery in individuals with AUD. The structure of this review follows a mechanism-based organization, concluding with translational and clinical perspectives on future research directions (Figure 1).

## 2. Ethanol Metabolism and Its Neurotoxic Intermediates

### 2.1. Oxidative Metabolism and Acetaldehyde

Ethanol is primarily metabolized via three enzymatic systems: alcohol dehydrogenase (ADH), catalase, and cytochrome P450 2E1 (CYP2E1). The vast majority of absorbed ethanol (approximately 90–98%) is oxidized in the liver, where high ADH and aldehyde dehydrogenase activities ensure systemic clearance, whereas only a minor fraction is metabolized directly within the brain [2,8,9,22]. Nevertheless, this quantitatively smaller intracerebral metabolism is critical for local neurotoxicity because it generates acetaldehyde and ROS in close proximity to neurons and glia. In the liver, ADH is the dominant oxidative pathway, but its expression in brain tissue is relatively low. In contrast, in neural tissue catalase and CYP2E1 constitute the major oxidative systems for ethanol [23].

Catalase, a heme-containing peroxisomal enzyme widely expressed in neurons and glia, uses hydrogen peroxide to oxidize ethanol to acetaldehyde. In vivo and pharmacological studies have demonstrated that brain catalase significantly contributes to local acetaldehyde formation and that inhibition of catalase decreases ethanol-derived acetaldehyde levels and attenuates several behavioural effects of ethanol [18,22,24]. Because catalase-dependent ethanol oxidation is driven by the availability of H_2_O_2_, its relative contribution is thought to increase under conditions of enhanced oxidative metabolism and high ethanol intake, thereby linking heavy drinking to intracerebral acetaldehyde production and region-specific neurotoxicity in the central nervous system [22,24].

CYP2E1 catalyzes the formation of acetaldehyde, a highly reactive and toxic metabolite that disrupts protein folding, interferes with intracellular signaling, and forms DNA and protein adducts [11,25]. This enzymatic activity is also a major source of ROS, leading to lipid peroxidation, mitochondrial damage, and oxidative DNA injury [8,12,26,27]. Notably, CYP2E1 has been localized to both the endoplasmic reticulum and mitochondria of neural cells, amplifying its neurotoxic potential via mitochondrial oxidative stress [18,23]. Importantly, as demonstrated by Zimatkin and Buben, significant ethanol oxidation occurs directly within the brain, supporting the concept that local acetaldehyde production contributes to region-specific neurotoxicity [22]. Interindividual variability in these metabolic pathways is substantial, as shown by Yamamoto et al., who demonstrated that genetic polymorphisms in alcohol dehydrogenase and aldehyde dehydrogenase significantly influence acetaldehyde accumulation and modify susceptibility to ethanol-induced toxicity [28]. Subsequent studies further detailed the enzymatic systems responsible for ethanol oxidation in neural tissue including catalase, CYP2E1, and ADH isoforms providing mechanistic support for brain-localized acetaldehyde generation during chronic alcohol exposure [24].

From a temporal perspective, acute low-to-moderate ethanol exposure typically produces short-lived increases in systemic and brain acetaldehyde that are efficiently cleared by aldehyde dehydrogenase, so that most behavioral effects are attributable to the parent compound [27]. In contrast, repeated or heavy drinking induces catalase and CYP2E1, can transiently saturate aldehyde dehydrogenase activity, and leads to higher and more sustained acetaldehyde levels in blood and brain [23,26]. Animal and human studies indicate that chronically alcohol-exposed subjects display increased basal acetaldehyde concentrations and exaggerated acetaldehyde peaks after a given ethanol dose, providing a biochemical basis for the greater neurotoxic burden associated with chronic alcohol use [27,28].

### 2.2. Salsolinol and Dopaminergic Toxicity

Acetaldehyde can react non-enzymatically with dopamine to form salsolinol, a tetrahydroisoquinoline derivative with strong neurotoxic properties [13,14]. In addition to this non-enzymatic pathway, Xiong et al. demonstrated that salsolinol synthase (Sal synthase) activity directly promotes the production of salsolinol and N-methyl-salsolinol, both of which exhibit potent cytotoxicity in dopaminergic cells [29]. Salsolinol has been implicated in the selective degeneration of dopaminergic neurons and is considered a contributing factor to alcohol-related Parkinsonism [13]. Both in vitro and in vivo studies have demonstrated that salsolinol impairs mitochondrial function, increases oxidative stress, and triggers apoptotic pathways in dopaminergic neurons [14,15]. Recent findings also suggest that salsolinol may promote inflammatory signaling and pyroptosis, further aggravating neuronal injury [20]. Wang et al. provided mechanistic confirmation of this process, showing that salsolinol activates NLRP3-dependent pyroptosis in dopaminergic neurons, thereby contributing to Parkinsonian-like pathology [30].

A similar distinction applies to salsolinol. Single episodes of ethanol intake generate only modest amounts of salsolinol, whereas chronic or binge-like exposure markedly increases salsolinol and N-methyl-salsolinol levels in dopaminergic regions such as the striatum and hypothalamus, as well as in biological fluids of alcohol-dependent subjects [31,32,33,34]. These elevations correlate with cumulative alcohol intake and may persist beyond acute exposure and into the early abstinence period, supporting the view that salsolinol is predominantly a marker and mediator of chronic rather than purely acute ethanol toxicity in dopaminergic circuits [33,34].

### 2.3. Non-Oxidative Pathways and Membrane-Targeting Metabolites

Beyond oxidative metabolism, ethanol undergoes non-oxidative biotransformation to yield fatty acid ethyl esters (FAEEs) and phosphatidylethanol (PEth), which accumulate in neural tissue [16,17]. Fatty acid ethyl esters (FAEEs) and phosphatidylethanol (PEth) are not only non-oxidative metabolites but also clinically relevant biomarkers of alcohol exposure. FAEEs, which accumulate in adipose tissue, liver, hair and meconium, have been used as long-term markers of chronic or prenatal alcohol exposure, particularly in forensic and perinatal settings [35,36]. In contrast, PEth formed in erythrocyte membranes is now widely adopted in clinical laboratories as a highly sensitive and specific blood biomarker of chronic and heavy alcohol intake, outperforming traditional markers such as γ-glutamyltransferase and carbohydrate-deficient transferrin in detecting recent regular drinking and monitoring abstinence. Thus, non-oxidative metabolites provide both mechanistic insight into ethanol toxicity and practical tools for the objective assessment of drinking patterns in patients with AUD [37,38].

These lipid-soluble metabolites integrate into cell membranes, altering membrane fluidity, impairing receptor function, and disrupting signal transduction [8]. Their presence in postmortem brain tissue has been proposed as a biomarker of chronic ethanol exposure [18]. Additionally, PEth is known to modulate phospholipase D signaling and interfere with lipid raft composition, further contributing to membrane-associated neurotoxicity [8].

### 2.4. Implications

**Figure 2:**
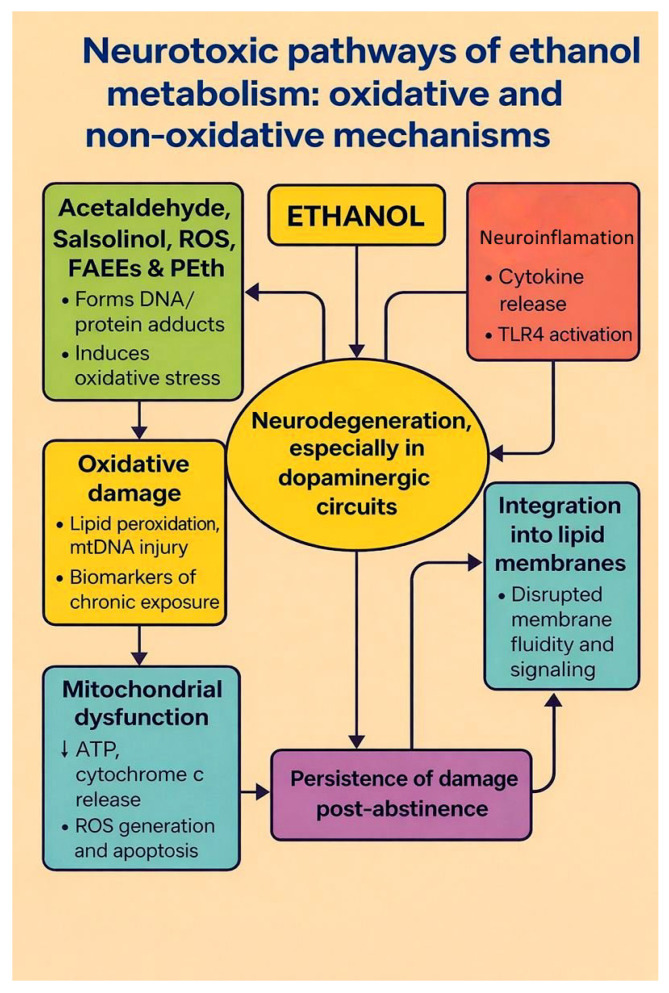
Neurotoxic pathways of ethanol metabolism: oxidative and non-oxidative mechanisms. The diagram summarizes how chronic ethanol exposure leads to neurodegeneration. Ethanol is converted to acetaldehyde, salsolinol, ROS, fatty acid ethyl esters (FAEEs) and phosphatidylethanol (PEth) (green box). These reactive intermediates form DNA and protein adducts and promote oxidative damage (lipid peroxidation and mitochondrial DNA injury; yellow box), which in turn contributes to mitochondrial dysfunction (blue box; ↓ ATP production, cytochrome c release, ROS-driven apoptosis). In parallel, ethanol and its metabolites activate neuroinflammatory signalling (cytokine release, TLR4 activation; red box) and integrate into lipid membranes, disrupting membrane fluidity and receptor signalling (turquoise box). All of these processes converge on neurodegeneration, especially in dopaminergic circuits (central oval) and contribute to the persistence of structural and functional brain damage even after abstinence (purple box). Arrows indicate the directional relationships between modules, and bidirectional arrows denote vicious cycles between metabolite formation, neuroinflammation and neurodegeneration. Created by the authors in Adobe Illustrator version 27.5, 2023 (created on 29 October 2025).

Taken together, ethanol metabolism yields a spectrum of reactive intermediates, including acetaldehyde, ROS, lipid peroxidation products, and non-oxidative metabolites, that converge on oxidative damage, organellar dysfunction, and apoptotic degeneration (Figure 2) [13,14,15,18]. These mechanistic insights are consistent with the broader metabolic framework proposed by Teschke [39], in which alcoholic organ injury is driven by the cumulative burden of ethanol and its metabolites on vulnerable cellular targets. In this framework, the balance between classical oxidative pathways (ADH, catalase, CYP2E1) and non-oxidative routes determines the generation of acetaldehyde, ROS, and bioactive lipids, which in turn initiate steatosis, inflammation, fibrogenesis, and cell death in alcoholic liver disease. Although originally formulated for hepatic pathology, the similar metabolic principles apply to the central nervous system: increased systemic and brain-local acetaldehyde formation, CYP2E1 induction, and mitochondrial oxidative stress provide a biochemical substrate for neuronal and glial injury, thereby linking drinking patterns and individual susceptibility to region-specific neurodegeneration.

## 3. Disruption of Protein Homeostasis in Neurons

### 3.1. Oxidative Protein Damage and ER Stress

Neurons critically rely on tightly regulated protein synthesis, folding, trafficking and degradation to sustain synaptic function and cellular homeostasis. Chronic alcohol exposure perturbs this balance by impairing both protein synthesis and clearance, leading to the accumulation of damaged or misfolded proteins and resulting in proteotoxic stress [16]. Ethanol and its metabolites cause oxidative modifications of cysteine and methionine residues, yielding aberrant protein structures that are cytotoxic and prone to aggregation [17].

A key consequence of protein misfolding is the induction of endoplasmic reticulum (ER) stress, which triggers the unfolded protein response (UPR) a compensatory signalling cascade aimed at restoring proteostasis. However, persistent or excessive activation of the UPR, particularly in metabolically compromised neurons, can initiate apoptotic signalling pathways [19]. Experimental models of alcohol exposure have shown elevated levels of ER stress markers, including CHOP, ATF4 and GRP78, in the brain, correlating with neuronal loss and impaired cognitive function [16,20].

Recent work has identified ferroptosis, an iron-dependent form of regulated cell death characterised by phospholipid peroxidation, as a salient mechanism in ethanol-induced neuronal injury. In a murine model of chronic intermittent alcohol exposure, Xu et al. [40] demonstrated increased iron accumulation, decreased GPX4 expression and marked neuronal damage that were prevented by ferrostatin-1. This emerging evidence suggests that ethanol-driven lipid peroxidation and impaired antioxidant defence may converge on ferroptotic pathways, thereby linking ER stress, ROS generation and lipid dysmetabolism into a unified neurodegenerative cascade.

### 3.2. Impairment of Protein Degradation Pathways

Chronic ethanol exposure also interferes with the ubiquitin–proteasome system (UPS), the principal pathway for the selective degradation of damaged, misfolded or short-lived regulatory proteins. In neurons, UPS activity is crucial for maintaining synaptic function, as it regulates the turnover of ion channels, neurotransmitter receptors and scaffold proteins at pre- and postsynaptic sites. Ethanol has been shown to inhibit proteasome catalytic activity and to alter the expression and post-translational modification of proteasome subunits, leading to the accumulation of polyubiquitinated proteins and insoluble aggregates in vulnerable brain regions [16,20]. These aggregates sequester components of the proteostasis network and disrupt intracellular signalling, thereby exacerbating cellular stress.

This proteasomal impairment closely resembles the pathogenic processes observed in classical neurodegenerative proteinopathies such as Alzheimer’s and Parkinson’s diseases, where insufficient clearance of aberrant proteins leads to progressive aggregation and neuronal death [1]. In the context of alcohol-induced neurotoxicity, accumulated misfolded proteins can include oxidatively modified cytoskeletal elements, synaptic proteins and mitochondrial components, which together compromise axonal transport, synaptic transmission and energy metabolism [16,40]. Moreover, proteasome dysfunction may further potentiate ER stress and ferroptotic pathways by allowing the persistence of oxidised and lipid-peroxidation–derived adducts, thereby integrating UPS failure into a broader cascade of proteotoxic and oxidative injury [20,40].

In parallel, autophagy, another major protein and organelle degradation pathway, is increasingly recognised as a critical target of ethanol toxicity. Under physiological conditions, macroautophagy and mitophagy eliminate damaged organelles and long-lived proteins, complementing UPS-mediated degradation. However, chronic ethanol exposure disrupts autophagic flux, for example, by impairing autophagosome–lysosome fusion or by dysregulating key regulators such as mTOR, Beclin-1 and LC3, which leads to the accumulation of dysfunctional mitochondria and toxic protein aggregates [7,35]. Rather than exerting a uniformly protective response, autophagy becomes insufficient or maladaptive in the chronically exposed brain, thereby amplifying proteotoxic stress and contributing to neuronal dysfunction and death [7,41]. Together, UPS inhibition and defective autophagy represent a convergent failure of protein quality control that is central to ethanol-induced neurodegeneration.

### 3.3. Chaperone Dysfunction and Loss of Proteostasis

Molecular chaperones constitute a third critical pillar of the proteostasis network. Heat shock proteins (HSPs), including HSP70, HSP90 and small HSPs, as well as ER-resident chaperones such as GRP78/BiP, supervise nascent polypeptide folding, prevent inappropriate protein–protein interactions and triage irreversibly damaged proteins toward UPS- or autophagy-mediated degradation. In neurons, which possess exceptionally high rates of protein turnover at synapses, chaperones are indispensable for maintaining functional connectivity under conditions of metabolic and oxidative stress.

Alcohol disrupts both the expression and stress-inducible upregulation of key chaperones. Experimental studies show that chronic ethanol exposure blunts the heat shock response, reduces basal HSP70 and HSP90 levels, and alters their ATPase-dependent folding activity [42]. In addition, oxidative modifications and acetaldehyde adduct formation on chaperone proteins further compromise their function, thereby limiting the cell’s ability to refold misfolded substrates or to deliver them efficiently to degradation pathways [17,42]. As a result, chaperone insufficiency synergises with proteasome inhibition and defective autophagy to promote the accumulation of toxic protein species and destabilise neuronal proteostasis.

Beyond protein folding, chaperones modulate key signalling pathways that determine cell fate. HSP90 and HSP70 interact with components of the apoptotic machinery and kinases such as Akt, influencing the balance between survival and death signalling, while GRP78 integrates ER stress responses with downstream UPR and apoptotic cascades [19,42]. Ethanol-induced chaperone dysfunction therefore not only impairs protein quality control but also removes important brakes on apoptotic and inflammatory signalling. Collectively, the combined impairment of folding, degradation and chaperone systems amplifies neuronal vulnerability to ethanol-induced stressors and establishes disruption of protein homeostasis as a central axis of alcohol-related neurodegeneration.

## 4. Mitochondrial Impairment and Bioenergetic Failure

Mitochondria play a vital role in ATP production, calcium buffering and apoptotic regulation, and are particularly abundant in neurons due to their high metabolic demand. Ethanol-induced mitochondrial dysfunction is therefore a central event in alcohol-related neurodegeneration [2,8,42]. In parallel with classical mitochondrial dysfunction, recent studies emphasise the role of altered mitochondrial dynamics in ethanol-mediated neurotoxicity. Specifically, excessive Drp1-mediated fission and suppressed mitofusin 2 (Mfn2) compromise mitochondrial network integrity and accelerate neuronal energy failure. Song et al. report that Nrf2/NQO1 signalling modulates ethanol-induced ferroptosis in cardiomyocytes, hinting at integrative mechanisms linking mitochondrial disintegration, redox imbalance and lipid peroxidation that likely extend to neural tissue [43].

### 4.1. Structural Damage and Cytochrome c Release

Ethanol exposure disrupts mitochondrial membrane potential, impairs electron transport chain (ETC) activity and reduces ATP synthesis [44]. A critical target is the voltage-dependent anion channel (VDAC), which regulates the exchange of ions and metabolites across the outer mitochondrial membrane. Ethanol or its metabolites inhibit VDAC function, leading to mitochondrial swelling, loss of membrane potential and release of cytochrome c, thereby triggering the intrinsic apoptotic cascade [18,33,44].

Recent conceptual advances highlight that VDAC is not merely a passive metabolite conduit but a dynamic regulator of mitochondrial homeostasis. VDAC functions as a key governor of mitochondrial permeability transitions and may act as an upstream actuator of ferroptotic signalling. These insights further underscore the vulnerability of ethanol-exposed neurons to VDAC-dependent bioenergetic collapse [45].

### 4.2. Oxidative Injury and Mitophagy Dysfunction

Mitochondrial CYP2E1, upregulated in alcohol-exposed neurons, generates excessive ROS during ethanol metabolism. The central role of alcohol-induced oxidative stress has long been established, as summarised by Wu and Cederbaum, who demonstrated that ethanol metabolism markedly increases free radical generation and promotes widespread oxidative injury [46]. These reactive species damage mitochondrial DNA, proteins and lipids, amplifying oxidative stress and accelerating mitochondrial decay [8,9].

Damaged mitochondria are typically targeted for degradation via mitophagy, a key quality control mechanism. However, chronic ethanol exposure impairs mitophagic efficiency, leading to the accumulation of dysfunctional mitochondria and further compromising neuronal viability [41]. These observations are consistent with updated biochemical analyses by Mailloux, who emphasised that mitochondrial ROS production is a dynamic and tightly regulated process that becomes profoundly dysregulated under toxic metabolic conditions such as chronic ethanol exposure [47].

### 4.3. Mitochondrial Dynamics and Energetic Failure

In addition to direct structural damage, ethanol disrupts the balance between mitochondrial fission and fusion. Studies show upregulation of the fission protein Drp1 and downregulation of the fusion protein Mfn2, resulting in mitochondrial fragmentation [48]. Consistent with this mechanism, Yan and Zhao demonstrated that acetaldehyde induces Drp1 phosphorylation and mitochondrial dysfunction via ROS elevation and intracellular Ca^2+^ dysregulation, reinforcing the contribution of ethanol metabolites to pathological mitochondrial fission [49]. Structural studies by Ugarte-Uribe et al. further revealed that Drp1 polymerisation stabilises highly curved mitochondrial membranes characteristic of constricted organelles, supporting its central role in pathological fission during ethanol exposure [50].

Fragmented mitochondria are metabolically less efficient and more susceptible to triggering pro-apoptotic signalling. Consistently, Tapia-Rojas et al. demonstrated that adolescent binge-like ethanol exposure induces profound mitochondrial impairment and bioenergetic deficits, further linking altered mitochondrial dynamics with long-term cognitive dysfunction [51]. Ultimately, the combination of impaired energy metabolism, elevated oxidative burden, defective quality control and dysregulated dynamics leads to bioenergetic collapse and synaptic dysfunction. Therapeutic strategies targeting mitochondrial integrity and function may therefore offer promising avenues for mitigating alcohol-induced neuronal injury.

## 5. Alcohol-Induced Neuroinflammation

### 5.1. Microglial Activation and TLR4 Signaling

Neuroinflammation represents a central mechanism of ethanol-induced neurotoxicity, contributing to both the initiation and progression of brain damage. Chronic alcohol exposure activates microglia, the brain’s resident immune cells, triggering the release of pro-inflammatory cytokines such as tumor necrosis factor-alpha (TNF-α), interleukin-1β (IL-1β) and interleukin-6 (IL-6) [4,5,19]. The intensity and regional pattern of microglial activation vary according to the dose, duration and developmental timing of ethanol exposure, with particular vulnerability noted in the hippocampus, cortex and cerebellum. Among the key molecular drivers of microglial activation is Toll-like receptor 4 (TLR4), which is upregulated in response to ethanol. Activation of TLR4 initiates downstream signaling through MyD88- and TRIF-dependent pathways, leading to NF-κB translocation and transcription of inflammatory mediators [5,19]. Notably, TLR4 knockout mice exhibit partial protection against alcohol-induced glial activation and neuronal loss, emphasizing the receptor’s pathogenic relevance [19].

Recent attention has focused on the concept of microglial “trained immunity”, a form of innate immune memory in which prior exposure to ethanol or other inflammatory stimuli induces long-lasting epigenetic and metabolic reprogramming of microglia [52,53,54]. In this primed state, microglia display exaggerated and prolonged production of pro-inflammatory cytokines and ROS (ROS) in response to subsequent challenges, even when alcohol is no longer present [55,56].

Ye et al. highlighted that such neuroimmune mechanisms are central to the pathophysiology of AUD, with microglial dysregulation tightly linked to neuronal and synaptic dysfunction [57]. Experimental models of chronic ethanol administration further demonstrate that microglial trained immunity is accompanied by upregulated TLR4 and chemokine signalling, increased recruitment of peripheral monocytes/macrophages into the brain and persistent alterations in resident microglia [57,58]. In line with this framework, Zhang and Luo identified MCP-1/CCR2-dependent chemokine signalling as a critical mediator of alcohol-induced microglial activation and neuronal injury, underscoring the importance of chemokine-driven immune dysregulation in AUD [59]. Consistent with these mechanisms, Li et al. demonstrated that adolescent ethanol exposure robustly activates microglia via TLR4 signalling and dysregulates the SUMO-specific protease SENP6, resulting in exacerbated neuroinflammation in the hippocampus. These findings emphasize the heightened vulnerability of the developing brain to TLR4-mediated neuroimmune activation [60].

### 5.2. Astrocytic Dysfunction and Excitotoxicity

Astrocytes, essential regulators of glutamate uptake and synaptic homeostasis, become functionally compromised during chronic ethanol exposure. Recent scoping and narrative reviews synthesise converging evidence that AUD is associated with profound alterations in astrocyte morphology, protein expression and signalling across multiple brain regions, including cortex, hippocampus and limbic structures [61,62,63]. Ethanol has been shown to downregulate the expression and/or impair the function of excitatory amino acid transporters (EAAT1/GLAST and EAAT2/GLT-1), leading to extracellular glutamate accumulation, loss of synaptic compartmentalisation and increased excitotoxic stress on neighbouring neurons [20,64,65,66,67,68,69].

Preclinical studies demonstrate that chronic and intermittent ethanol exposure reduces GLT-1 levels in key reward and stress-related regions, such as the prefrontal cortex, nucleus accumbens and hypothalamic nuclei, thereby promoting pathological hyperglutamatergic states and vulnerability to excitotoxic damage [67,69,70].

Ethanol-exposed astrocytes also exhibit morphological atrophy, reduced coverage of synapses and blood vessels, impaired K^+^ buffering and altered gap-junction coupling, further destabilising local microcircuits and amplifying neuronal vulnerability [11,20,61,62,71].

At the cellular level, Luo et al. demonstrated that ethanol differentially affects cortical astrocytes and neurons, with astrocytes showing pronounced disturbances in calcium signalling, redox balance and trophic support, changes that secondarily enhance glutamate-mediated neurotoxicity [64]. Complementary work revealed that ethanol-induced changes in transcription factor and extracellular matrix-related gene expression in astrocytes alter their ability to regulate synaptogenesis and protect neurons, thereby linking astrocytic gene programmes to excitotoxic injury [65,72]. Collectively, these findings indicate that astrocytic dysfunction is not merely a bystander consequence of neuronal damage but a primary driver of glutamatergic dysregulation and excitotoxic neurodegeneration in AUD.

### 5.3. Glial Priming and Persistent Inflammation

Importantly, the neuroinflammatory effects of alcohol are not transient. Repeated ethanol exposure leads to glial priming, a state of sensitisation in which microglia exhibit exaggerated responses to subsequent stimuli even in the absence of ongoing alcohol intake [4,20]. This sustained reactivity may underlie long-term cognitive and emotional impairments observed in individuals with AUD, even during abstinence. Emerging therapeutic strategies targeting neuroinflammatory signalling such as TLR4 antagonists, NF-κB inhibitors and modulators of microglial activation have shown promise in preclinical models and may offer effective adjunctive treatments for AUD.

## 6. Calcium Dysregulation and Synaptic Dysfunction

### 6.1. Ethanol-Induced Calcium Influx and Signaling

Intracellular calcium (Ca^2+^) functions as a crucial second messenger that regulates neurotransmitter release, gene transcription, synaptic plasticity and cell survival [41,73]. Ethanol interferes with calcium homeostasis by modulating the activity of calcium-permeable ion channels and receptors, including NMDA and AMPA glutamate receptors, voltage-gated calcium channels (VGCCs) and intracellular stores such as the endoplasmic reticulum (ER) [14,47,48,59].

One of the earliest observed effects of ethanol on synaptic physiology is the potentiation of NMDA receptor-mediated calcium influx, particularly in the developing brain [42]. Elevated intracellular Ca^2+^ activates Ca^2+^/calmodulin-dependent protein kinase II (CaMKII) and protein kinase C (PKC), promoting phosphorylation of cytoskeletal proteins and disruption of synaptic architecture. These calcium-dependent mechanisms align with recent findings by Lim et al., showing that ethanol-activated CaMKII triggers excessive Drp1-mediated mitochondrial fission and promotes JNK1-dependent activation of the NLRP3 inflammasome, ultimately leading to neuronal apoptosis. This mechanistic axis provides a direct link between Ca^2+^ dysregulation, mitochondrial fragmentation and inflammatory cell death [74].

### 6.2. ER Calcium Depletion and Unfolded Protein Response

Chronic ethanol exposure also stimulates the release of calcium from the ER via inositol 1,4,5-trisphosphate receptors (IP_3_Rs) and ryanodine receptors (RyRs), leading to ER calcium depletion and the induction of ER stress. The resulting activation of the unfolded protein response (UPR) may initially serve a protective role, but persistent activation contributes to apoptotic signalling cascades [16,44].

### 6.3. Mitochondrial Calcium Overload and Neuronal Death

Mitochondria buffer cytosolic calcium to prevent toxicity; however, prolonged ethanol exposure causes mitochondrial calcium overload, which triggers the opening of the mitochondrial permeability transition pore (mPTP). In addition to mitochondrial overload, Shaidullov et al. demonstrated that the ethanol metabolite acetic acid activates BK channels in a pH-dependent manner and disrupts calcium oscillations and secretory granule exocytosis, revealing an additional mechanism by which ethanol metabolites destabilise neuronal Ca^2+^ homeostasis [75]. This sequence of events results in collapse of the mitochondrial membrane potential, elevated production of ROS and the release of pro-apoptotic factors such as cytochrome c [33,44].

**Figure 3:**
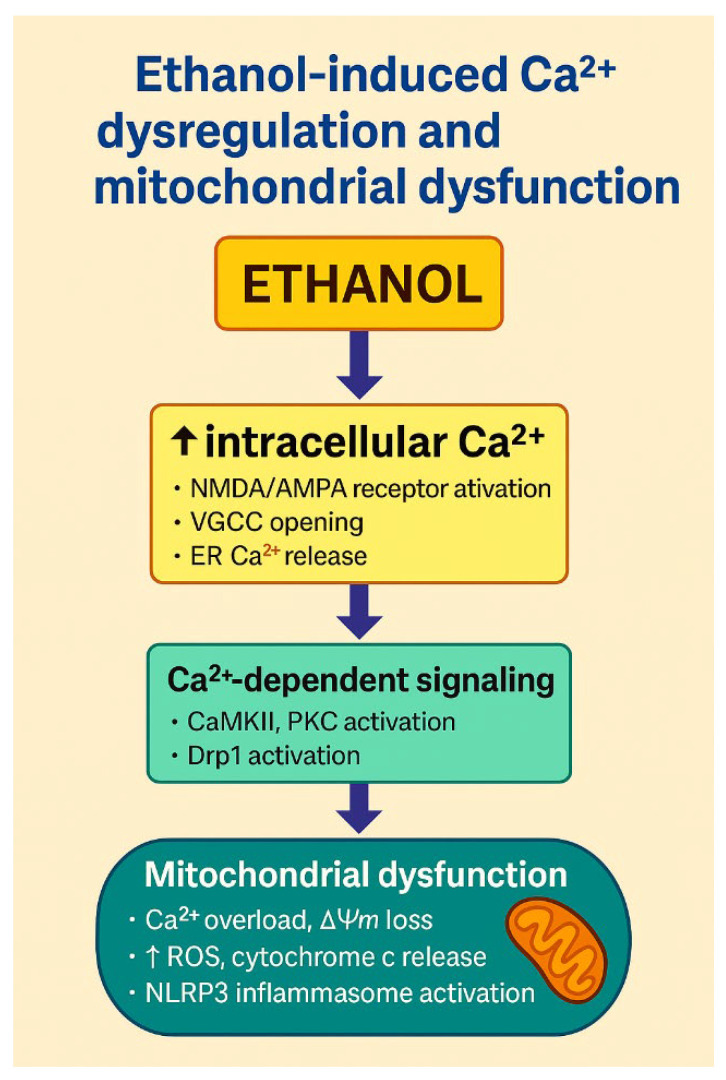
Ethanol-induced Ca^2+^ dysregulation and mitochondrial dysfunction. The schematic illustrates how ethanol disrupts neuronal Ca^2+^ homeostasis and promotes mitochondrial injury. Ethanol exposure leads to ↑ intracellular Ca^2+^, in part through enhanced activation of NMDA/AMPA glutamate receptors, opening of voltage-gated calcium channels (VGCCs) and release of Ca^2+^ from endoplasmic reticulum stores. Elevated Ca^2+^ activates Ca^2+^-dependent signalling pathways, including CaMKII and PKC, and promotes Drp1-mediated mitochondrial fission. Mitochondrial Ca^2+^ overload results in loss of mitochondrial membrane potential (ΔΨm), increased generation of ROS, cytochrome c release and activation of the NLRP3 inflammasome, culminating in neuronal apoptosis. Together, these processes link ethanol-induced Ca^2+^ dysregulation with mitochondrial dysfunction and inflammatory cell death. Created by the authors in Adobe Illustrator version 27.5, 2023 (created on 30 October 2025).

Moreover, the interplay between oxidative stress and impaired autophagy is increasingly recognised as a driver of alcohol-mediated neuronal damage. Ruiter-Lopez et al. (2025) showed that chronic ethanol consumption disrupts mitophagy/autophagy pathways, linking elevated ROS to persistent neuronal injury [23]. Disturbed calcium homeostasis and mitochondrial dysfunction thus form a vicious feedback loop that drives synaptic degeneration and neuronal loss. This loop is further exacerbated by concurrent oxidative and inflammatory stressors, contributing to progressive cognitive impairment (Figure 3).

## 7. Epigenetic Remodeling in Alcohol-Induced Neurotoxicity

Chronic alcohol exposure drives long-lasting alterations in gene expression that cannot be explained solely by changes in DNA sequence [4,6,33]. Converging evidence from human and animal studies indicates that AUD is accompanied by widespread epigenetic remodelling, including DNA methylation changes, histone post-translational modifications and altered non-coding RNA profiles in brain and peripheral tissues [4,6,33,76,77,78]. These epigenetic adaptations affect stress-responsive pathways, reward circuitry, neuroimmune signalling and synaptic plasticity, thereby contributing to progressive neurodegeneration and behavioural [77,78]. Recent reviews have synthesised these findings, highlighting that alcohol-induced epigenetic modifications are detectable across development (prenatal, adolescent and adult exposure), show region- and cell type-specific patterns and may serve as potential biomarkers and therapeutic targets in AUD [79,80,81].

### 7.1. DNA Methylation

Ethanol disrupts both global and gene-specific patterns of DNA methylation in the brain [33]. Epigenome-wide association studies in patients with AUD and in alcohol-dependent animal models have identified differentially methylated CpG sites in genes involved in synaptic transmission, neuroimmune signalling, myelination and stress response, with some alterations correlating with disease severity and treatment outcome [77,81]. Multi-cohort analyses have validated differential methylation of HECW2 and GDAP1 as robust epigenetic markers of AUD in blood and brain, and have revealed sex-specific effects and partial reversibility with abstinence [82]. In parallel, developmental studies show that prenatal and adolescent alcohol exposure alters methylation of genes controlling the hypothalamic–pituitary–adrenal axis, circadian rhythm, neurotrophic factors and glutamatergic receptors, thereby predisposing to long-term neurobehavioral deficits and increased vulnerability to AUD later in life [79,80]. At the promoter level, chronic ethanol exposure reduces global 5-methylcytosine levels while selectively inducing hypomethylation in promoters of pro-inflammatory genes such as IL1B and TNF, thereby enhancing their transcriptional activity [33]. These modifications are driven, at least in part, by impaired activity of DNA methyltransferases (DNMTs), especially DNMT1 and DNMT3a, both essential for maintaining neuronal identity and genomic stability [33,73]. Complementary evidence from Zhang et al. shows that ethanol-induced alterations in DNA methylation upregulate tissue plasminogen activator expression in astrocytes, illustrating how epigenetic dysregulation contributes to gliocentric mechanisms of neurotoxicity [65]. Together, these data support the view that ethanol-induced DNA methylation changes are not only correlates of exposure but also mechanistic contributors to altered neuronal and glial function.

### 7.2. Histone Modifications

Alcohol-induced neurotoxicity is also associated with profound disturbances in chromatin structure mediated by histone post-translational modifications [6]. Experimental models of developmental and adult ethanol exposure demonstrate altered activity of histone acetyltransferases (HATs) and histone deacetylases (HDACs), leading to region-specific changes in histone acetylation (e.g., H3K9ac, H4K12ac) and methylation (e.g., H3K4me3, H3K9me2/3) at promoters of genes involved in synaptic plasticity, neuroinflammation and cell-survival pathways [6,41,83].

Subbanna and colleagues showed that neonatal ethanol exposure reduces histone acetylation at *Bdnf* and other plasticity-related loci in the hippocampus, resulting in persistent learning and memory deficits that can be partially reversed by HDAC inhibition [83]. More recently, Kawecka et al. reported that in patients with AUD, the *GDAP1* locus is characterised by altered H3K4me3 levels that normalise after abstinence-based therapy, suggesting that histone marks at specific genes may act as dynamic indicators of treatment response [84].

Integrating these findings, current reviews emphasise that histone-based mechanisms provide a critical interface between ethanol-induced signalling cascades, chromatin accessibility and long-term changes in gene expression [1,6].

### 7.3. microRNAs and Post-Transcriptional Regulation

Ethanol alters the expression of numerous brain-enriched microRNAs (miRNAs) that fine-tune networks controlling synaptic plasticity, neurogenesis, neuroinflammation and glial activation [4,17,18,19]. Profiling studies of human alcoholic brain tissue and rodent models have identified consistent changes in miRNAs regulating neurotrophic factors (e.g., miR-132, miR-206), glutamatergic and GABAergic transmission (miR-9, miR-181a) and innate immune signalling (miR-155, miR-146a) [17,18,19].

In particular, downregulation of miR-132, miR-124 and miR-9 has been linked to enhanced inflammation, loss of neuronal resilience and microglial priming, as these miRNAs normally repress pro-apoptotic and pro-inflammatory targets; their suppression therefore amplifies ethanol-induced neuropathology [4,17]. Dysregulated miRNAs can both directly modulate neuronal targets and indirectly shape the epigenetic landscape by targeting DNMTs, HDACs and other chromatin regulators [17,20]. In developmental models, prenatal alcohol exposure reprogrammes miRNA profiles in cortex, hippocampus and hypothalamus, contributing to altered corticosterone signalling, circadian gene expression and long-lasting cognitive impairment [11,21]. Thus, miRNAs act as key post-transcriptional mediators through which ethanol exposure becomes embedded in neural circuits, and their persistent dysregulation amplifies ethanol-induced neuropathology.

## 8. Apoptotic Pathways and PI3K/Akt/mTOR Dysregulation

Apoptosis plays a pivotal role in alcohol-induced neurodegeneration, serving as the final common pathway integrating upstream molecular insults. Chronic ethanol exposure activates both the intrinsic (mitochondrial) and extrinsic (death receptor-mediated) apoptotic cascades, ultimately converging on the executioner caspases-3 and -9 [44,48].

### 8.1. Intrinsic Apoptotic Cascade

The intrinsic pathway is initiated by mitochondrial dysfunction, oxidative stress and pro-apoptotic protein signalling. Ethanol promotes the translocation of the pro-apoptotic effector Bcl-2–associated X protein (Bax) to the outer mitochondrial membrane, facilitating mitochondrial outer membrane permeabilization (MOMP) and release of cytochrome c [44]. This triggers apoptosome formation and activation of caspase-dependent proteolysis, leading to chromatin condensation and DNA fragmentation. The universal relevance of these apoptotic mechanisms has long been recognised in alcohol-related pathology, with Ockner demonstrating that chronic ethanol exposure activates conserved mitochondrial apoptotic pathways across organ systems, highlighting their central role in ethanol-induced cellular injury [85].

Further linking inflammation to apoptotic signalling, Qin et al. demonstrated that chronic alcohol exposure induces TRAIL-mediated neuronal death, establishing a mechanistic bridge between sustained neuroinflammation and engagement of mitochondrial apoptotic machinery in AUD [71].

### 8.2. Suppression of PI3K/Akt/mTOR Survival Signaling

Parallel to apoptotic activation, ethanol impairs survival pathways. The PI3K/Akt/mTOR signalling axis is crucial for neuronal survival, growth and protein synthesis. Ethanol suppresses Akt phosphorylation and mTOR activity, thereby enhancing susceptibility to apoptosis and inhibiting autophagy-mediated clearance of damaged proteins [41,48]. Ethanol also disrupts downstream effectors such as GSK-3β and BAD, removing key anti-apoptotic restraints. Supporting these observations, Sangaunchom and Dharmasaroja demonstrated that caffeine potentiates ethanol-induced neurotoxicity by further inhibiting the mTOR/p70S6K/4E-BP1 axis in neuronal cells, underscoring the central role of mTOR dysregulation in ethanol-related apoptotic vulnerability [66].

### 8.3. BDNF Downregulation and Synaptic Vulnerability

Ethanol-induced reductions in brain-derived neurotrophic factor (BDNF) further compromise neuronal survival. BDNF, acting mainly through TrkB receptors, supports dendritic growth, synaptic plasticity and anti-apoptotic signalling in cortical and limbic circuits. Experimental models of adolescent and adult ethanol exposure demonstrate that repeated alcohol administration decreases BDNF expression and signalling in the hippocampus, prefrontal cortex and amygdala, which is associated with impaired neurogenesis, dendritic spine loss and increased anxiety- and depression-like behaviour; pharmacological or genetic restoration of BDNF signalling can attenuate alcohol intake and partially rescue these structural and behavioural deficits [86,87].

Clinical observations align with these mechanistic data. Beyond individual cohort studies such as that of Martín-González et al., which reported markedly reduced circulating BDNF levels in patients with alcoholism [88], recent systematic and meta-analytic work indicates that individuals with AUD generally exhibit lower peripheral BDNF concentrations than healthy controls, with partial normalisation during abstinence and potential prognostic value for cognitive decline and relapse risk [89,90]. These converging preclinical and clinical findings support the view that chronic ethanol exposure impairs neurotrophic support and heightens synaptic and cognitive vulnerability through sustained disruption of BDNF–TrkB signalling.

**Figure 4:**
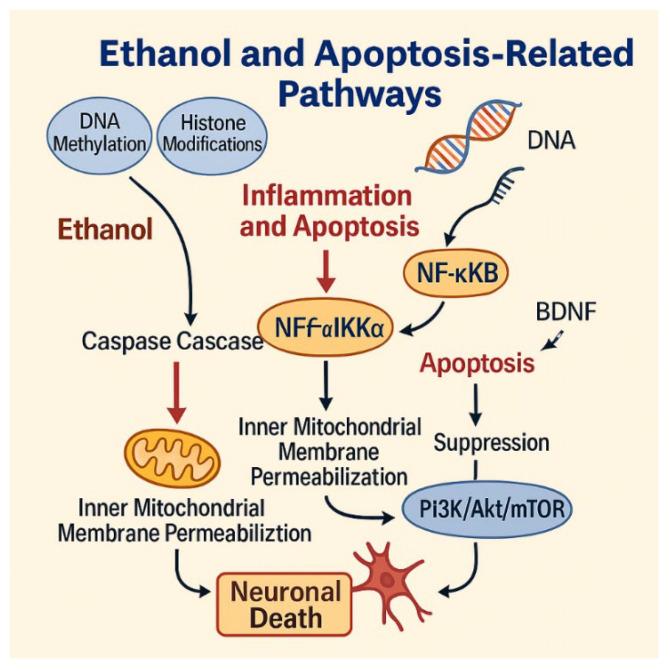
Ethanol-induced apoptotic pathways leading to neuronal death. Ethanol triggers epigenetic modifications, caspase activation, and mitochondrial dysfunction, while suppressing the PI3K/Akt/mTOR survival pathway and BDNF signaling. These converging mechanisms promote neuronal death. Figure created by the authors using Adobe Illustrator (version 27.5).

Targeting apoptotic and survival signalling nodes, including pharmacological activation of PI3K/Akt/mTOR or strategies aimed at restoring BDNF expression or TrkB activation, therefore offers promising neuroprotective avenues in alcohol-related CNS disorders [41,48,86] (Figure 4).

Recent studies have begun to validate this concept experimentally. In preclinical models, activation of the PI3K/Akt/mTOR pathway by BDNF mimetics, PI3K agonists or mTOR modulators reduces neuronal apoptosis, improves mitochondrial bioenergetics and enhances synaptic plasticity [66,88,91]. Agents such as 7,8-dihydroxyflavone (a TrkB/BDNF receptor agonist) and IGF-1 analogues promote Akt phosphorylation and protect hippocampal neurons against ethanol toxicity, while inhibition of GSK-3β, a downstream target negatively regulated by Akt, mitigates oxidative stress and neuronal loss [88].

Although clinical data are still limited, preliminary findings suggest that compounds enhancing BDNF–PI3K/Akt/mTOR activity, including certain antidepressants (SSRIs) and physical exercise interventions, correlate with improved neurocognitive outcomes and partial recovery of white matter integrity in individuals with AUD [91,92]. Together, these results indicate that pharmacological or lifestyle-based stimulation of the PI3K/Akt/mTOR axis represents a promising neuroprotective approach worthy of further translational investigation.

## 9. Alcohol-Induced White Matter Injury and Oligodendrocyte Vulnerability

Chronic alcohol exposure produces marked alterations in white matter architecture, reflecting detrimental effects on oligodendrocytes, the primary myelinating cells of the central nervous system. Oligodendrocytes are metabolically demanding cells that rely heavily on mitochondrial integrity and lipid homeostasis to sustain myelin synthesis. Ethanol-induced oxidative stress, mitochondrial dysfunction and lipid peroxidation significantly impair oligodendrocyte survival and disrupt myelin maintenance [17,18,91]. Moreover, alcohol interferes with oligodendrocyte precursor cell (OPC) proliferation and differentiation, limiting the capacity for myelin regeneration.

Neuroinflammatory signalling further exacerbates white matter injury. Microglial activation and cytokine release (TNF-α, IL-1β, IL-6) create an unfavourable milieu for oligodendrocyte survival, while astrocytic connexin dysfunction contributes to dysregulated glia–glia communication and reduced trophic support [4,17,91]. These convergent mechanisms promote myelin thinning, axonal conduction deficits and cognitive slowing commonly observed in individuals with AUD.

Emerging evidence suggests that ethanol exposure disrupts OPC maturation programmes, thereby impairing remyelination capacity [92]. Ethanol-induced activation of Wnt/β-catenin and Notch signalling has been implicated in arresting OPC differentiation, with consequent myelin thinning and white matter loss. Because white matter integrity is a critical determinant of cognitive and processing-speed deficits in AUD, integrating OPC pathology into the mechanistic narrative substantially enhances biological depth and translational relevance [93]. Given the essential role of white matter integrity in cognitive and emotional processing, targeting oligodendrocyte health may represent a promising therapeutic strategy for mitigating alcohol-related neurodegeneration. A synthetic overview of the main mechanisms described in Section 2, Section 3, Section 4, Section 5, Section 6, Section 7, Section 8 and Section 9 is provided in Table 1.

## 10. GABAergic Dysregulation in Chronic Alcohol Exposure

In addition to glutamatergic and dopaminergic disruptions, ethanol profoundly alters GABAergic signalling. Acute low-to-moderate alcohol exposure enhances GABA_A receptor activity and potentiates inhibitory tone, producing sedative and anxiolytic effects. In contrast, chronic heavy or binge drinking induces compensatory downregulation of GABA_A receptor subunits, altered receptor trafficking, and reduced inhibitory neurotransmission, culminating in neuronal hyperexcitability and heightened vulnerability to excitotoxic neuronal damage [42,76].

Beyond the GABAergic system, chronic alcohol use also remodels glutamatergic, dopaminergic, serotonergic, noradrenergic, and striatal cholinergic signalling, with dose- and pattern-dependent effects summarised in Table 2. In particular, recent findings indicate that ethanol modulates atypical GABA_A receptors on striatal cholinergic interneurons in the nucleus accumbens, thereby influencing dopamine release and reward processing [65]. This mechanism reinforces alcohol-seeking behaviour and contributes to long-term synaptic instability.

Moreover, reduced inhibitory GABAergic control can shift the excitation–inhibition balance toward hyperexcitability, thereby facilitating glutamate-driven Ca^2+^ loading and downstream mitochondrial stress [94,95]. Collectively, these adaptations shift the excitation–inhibition balance toward hyperexcitability, contributing to withdrawal-related neurobehavioral symptoms and increased vulnerability to excitotoxic neuronal damage.

## 11. Sex Differences in Alcohol-Induced Neurotoxicity

Biological sex is an important determinant of vulnerability to alcohol-induced brain injury. Epidemiological and experimental studies indicate that men generally consume greater absolute amounts of ethanol and engage more frequently in heavy or binge drinking, whereas women develop neurocognitive deficits, white matter deterioration and neuroinflammatory activation after shorter durations or lower quantities of alcohol exposure [19,91]. Thus, observed sex differences in alcohol-induced neurotoxicity reflect both differences in exposure patterns (dose, frequency and duration of intake) and intrinsic biological susceptibility. An overview of sex-specific vulnerabilities is provided in Table 3.

First, women experience higher blood alcohol concentrations (BAC) due to reduced gastric alcohol dehydrogenase activity and differences in body composition [28]. Second, oestrogen amplifies neuroinflammation by potentiating TLR4–NFκB signalling and increasing cytokine production [1,11,20]. Conversely, testosterone exerts partial neuroprotective effects by modulating oxidative pathways and supporting PI3K/Akt survival signalling [41,96].

Sex-dependent differences in mitochondrial resilience, calcium homeostasis and epigenetic remodelling, including differential expression of miRNAs and histone-modifying enzymes, further contribute to divergent neurotoxic outcomes [4,33]. Recognition of these sex-specific mechanisms is essential for developing personalised therapeutic strategies and designing translationally relevant preclinical models. These differences underscore the importance of incorporating sex-balanced experimental designs and conducting stratified clinical analyses.

## 12. Neuroprotective and Therapeutic Strategies in Alcohol-Induced Neurotoxicity

The mechanistic insights outlined in this review point to several converging targets for neuroprotective intervention in alcohol-induced brain injury. Rather than acting on a single pathway, effective strategies will likely need to address oxidative stress, mitochondrial dysfunction, neuroinflammation, calcium dysregulation, epigenetic remodelling and impaired neurotrophic signalling in an integrated manner [3,23,48].

### 12.1. Targeting Oxidative Stress and Mitochondrial Dysfunction

Given the central role of reactive oxygen and nitrogen species (ROS/RNS) and mitochondrial failure in ethanol-induced neurotoxicity, antioxidant and mitochondria-directed therapies have attracted substantial interest [8,10,23,46,47]. N-acetylcysteine and glutathione donors aim to restore cellular redox balance and limit lipid peroxidation, thereby reducing damage to membranes, mitochondrial DNA and respiratory chain complexes [10,46]. Likewise, mitochondrial stabilisers such as coenzyme Q10, MitoQ and SS-peptides support electron transport chain function, help maintain mitochondrial membrane potential and attenuate cytochrome c release and caspase activation [23,47,51]. Preclinical models of chronic ethanol exposure consistently show that these interventions decrease ROS generation, improve mitochondrial bioenergetics and partially preserve synaptic and cognitive function [8,23,51].

### 12.2. Modulating Neuroinflammation and Microglial Activation

Persistent activation of microglia and astrocytes, driven by TLR4–NFκB signalling and chemokine pathways (e.g., MCP-1/CCR2), is a major contributor to alcohol-related neurodegeneration [1,20,57,59,60]. Glial-targeted anti-inflammatory agents such as minocycline, TLR4 antagonists and CCR2/CCR5 inhibitors have been shown to reduce pro-inflammatory cytokine release, limit peripheral immune-cell recruitment and protect synapses in experimental models of chronic ethanol exposure [11,57,58]. By dampening microglial “trained immunity” and interrupting the feed-forward loop between oxidative stress and neuroinflammation, these compounds may help stabilise neuronal networks and slow both white- and grey-matter degeneration [11,57,58,59].

### 12.3. Stabilising Calcium Homeostasis and Limiting Excitotoxicity

Calcium dysregulation represents a key point of convergence for glutamatergic overactivation, mitochondrial dysfunction and GABAergic impairment [44,49,74]. In addition, pharmacological approaches that stabilise GABAergic signalling may alleviate withdrawal symptoms and help restore excitation–inhibition balance, thereby indirectly reducing excitotoxic neuronal injury.

Pharmacological strategies aimed at stabilising calcium dynamics including NMDA receptor antagonists (e.g., memantine), voltage-gated calcium channel (VGCC) blockers and modulators of endoplasmic reticulum (ER) stress have demonstrated neuroprotective effects in animal models of alcohol exposure [44,49,74]. These approaches reduce pathological Ca^2+^ influx, prevent mitochondrial Ca^2+^ overload and limit downstream activation of apoptotic cascades [49,74]. Although clinical data remain limited, such agents are promising candidates for adjunctive treatment in patients with alcohol use disorder who exhibit cognitive impairment or high risk of withdrawal-related neurotoxicity [44].

### 12.4. Epigenetic Therapeutic Strategies

Because many ethanol-induced epigenetic changes are reversible, they represent attractive targets for disease-modifying interventions [11,23,48,60,78]. Preclinical studies indicate that pharmacological modulation of DNA methyltransferases (DNMTs) and histone deacetylases (HDACs) can normalize aberrant DNA methylation and histone acetylation at promoters of neuroprotective genes, restore BDNF signalling, reduce neuroinflammation, and improve cognitive performance after developmental or adult ethanol exposure [11,65,83]. HDAC inhibitors (e.g., valproic acid, butyrate, and suberoylanilide hydroxamic acid) and other compounds enhancing histone acetylation have shown particular promise in rescuing ethanol-induced synaptic and behavioural deficits in rodent models [11,16,83].

Emerging evidence further suggests that miRNA-based approaches (miRNA mimics or antagomirs) may correct maladaptive post-transcriptional regulation and rebalance stress- and reward-related gene networks, in part through modulation of microglia-derived extracellular vesicles and neuroimmune signalling [11,12,23,48,65,83].

In parallel, lifestyle-based interventions, including physical exercise, environmental enrichment, and sustained abstinence, may partially reverse alcohol-related epigenetic marks at specific loci (e.g., GDAP1) and are associated with improvements in neurocognitive outcomes [77,84]. Collectively, translational reviews propose epigenetic modifiers, alone or in combination with established pharmacotherapies, as a novel class of adjuvant treatments aimed at reducing relapse risk and attenuating alcohol-related neurodegeneration [53,78,79].

### 12.5. Enhancing Neurotrophic and Pro-Survival Signalling

Disruption of PI3K/Akt/mTOR survival pathways and downregulation of BDNF represent crucial mechanisms by which ethanol promotes neuronal apoptosis and synaptic loss [7,44,48,88,96]. Agents that enhance PI3K/Akt/mTOR signalling or directly augment BDNF–TrkB activity are therefore being explored as neuroprotective candidates [48,66,96]. Experimental data show that pharmacological or genetic restoration of BDNF expression mitigates dendritic spine loss, improves hippocampal plasticity and reduces alcohol intake in animal models, while alterations in circulating BDNF levels have been reported in patients with alcoholism [88].

Activation of PI3K/Akt/mTOR signalling, or prevention of its inhibition by ethanol and related stressors, supports neuronal survival, autophagic clearance of damaged proteins and metabolic resilience [7,66,96]. Lifestyle-based interventions such as sustained abstinence, physical exercise and environmental enrichment have also been associated with improvements in neurocognitive outcomes and partial recovery of white matter integrity in alcohol-related brain damage [91,92,93], suggesting that combined behavioural and pharmacological approaches may yield synergistic benefits.

### 12.6. Multimodal and Translational Considerations

Given the multifactorial nature of alcohol-induced neurotoxicity, it is unlikely that single-target interventions will provide complete protection [3,21,23,48]. Combination strategies that integrate antioxidant, anti-inflammatory, epigenetic and neurotrophic approaches, together with approved medications for AUD and structured psychosocial interventions, may offer the greatest potential for preserving brain structure and function [11,21,48].

Future clinical trials should therefore consider multimodal designs, stratification by sex, age and pattern of alcohol use, and incorporation of neuroimaging and molecular biomarkers to monitor treatment response and neurobiological recovery [3,21,23,48]. A structured overview of the main therapeutic strategies and their molecular targets is provided in Table 4.

## 13. Limitations of Current Research and Translational Gaps

Although substantial progress has been made in elucidating alcohol-induced neuropathology, significant translational barriers remain. Most preclinical models fail to fully replicate the chronicity, behavioural complexity and comorbid metabolic and psychiatric conditions observed in human AUD [3,11]. Longitudinal biomarkers capable of predicting individual susceptibility to alcohol-related neurodegeneration are limited, with few validated molecular or imaging-based indicators available.

Mechanistic studies frequently examine isolated pathways of oxidative stress, mitochondrial impairment, neuroinflammation despite abundant evidence that ethanol toxicity emerges from highly interconnected signalling networks. Multi-omic analyses integrating transcriptomic, epigenetic, proteomic and metabolic data remain comparatively rare [5,6,33]. Furthermore, many promising therapeutic agents (e.g., HDAC inhibitors, DNMT modulators, mitochondrial stabilisers) have shown success only in rodent models, with few advancing into early-phase clinical trials [41,48].

Critical gaps also include insufficient analysis of sex differences, incomplete evaluation of white matter changes and limited focus on oligodendrocyte biology. In humans with chronic AUD, de la Monte and Tong (2025) demonstrate marked alterations in cerebellar white matter associated with oligodendrocyte dysfunction and impaired insulin/IGF-1 signalling [93]. These data emphasise the translational relevance of white matter pathology and glial mechanisms in alcohol-related brain degeneration. Addressing these issues is essential for developing targeted, mechanism-informed clinical interventions.

## 14. Conclusions and Future Perspectives

Chronic alcohol exposure exerts multifaceted neurotoxic effects that span metabolic, structural, inflammatory and epigenetic domains. These effects converge on a set of core homeostatic disruptions impaired protein folding, mitochondrial dysfunction, calcium dysregulation and aberrant gene expression that collectively undermine synaptic function, neuroplasticity and cellular survival. Notably, many of these disturbances are interconnected and self-perpetuating, forming a vicious cycle in which oxidative stress promotes neuroinflammation, which in turn exacerbates mitochondrial failure and apoptosis [4,41,44].

Elucidating these pathophysiological mechanisms provides a critical framework for the development of targeted therapeutic strategies. Antioxidants and mitochondrial stabilisers such as coenzyme Q10 and MitoQ have shown potential in reducing ROS-mediated injury, while HDAC inhibitors and DNA methyltransferase modulators are being explored to reverse maladaptive epigenetic changes [32,41]. Glial-targeted anti-inflammatory compounds—such as minocycline and TLR4 antagonists may attenuate neuroimmune activation. Calcium channel blockers and NMDA receptor antagonists could help restore ionic homeostasis and reduce excitotoxic burden [20,44]. Furthermore, enhancing PI3K/Akt/mTOR signalling and restoring BDNF expression represent promising neuroprotective strategies that warrant further preclinical and clinical investigation [41,48].

Future research should prioritise the identification of reliable biomarkers genetic, transcriptomic or imaging-based that predict individual susceptibility to alcohol-induced neurodegeneration and help stratify patients for personalised intervention. Given the systemic and multifactorial nature of ethanol neurotoxicity, integrated therapeutic approaches combining anti-inflammatory, metabolic, epigenetic and neurotrophic agents may yield the most favourable outcomes. Importantly, pharmacological strategies should be complemented by behavioural therapies and cognitive rehabilitation to enhance neurofunctional recovery and reduce relapse risk.

**Figure 5:**
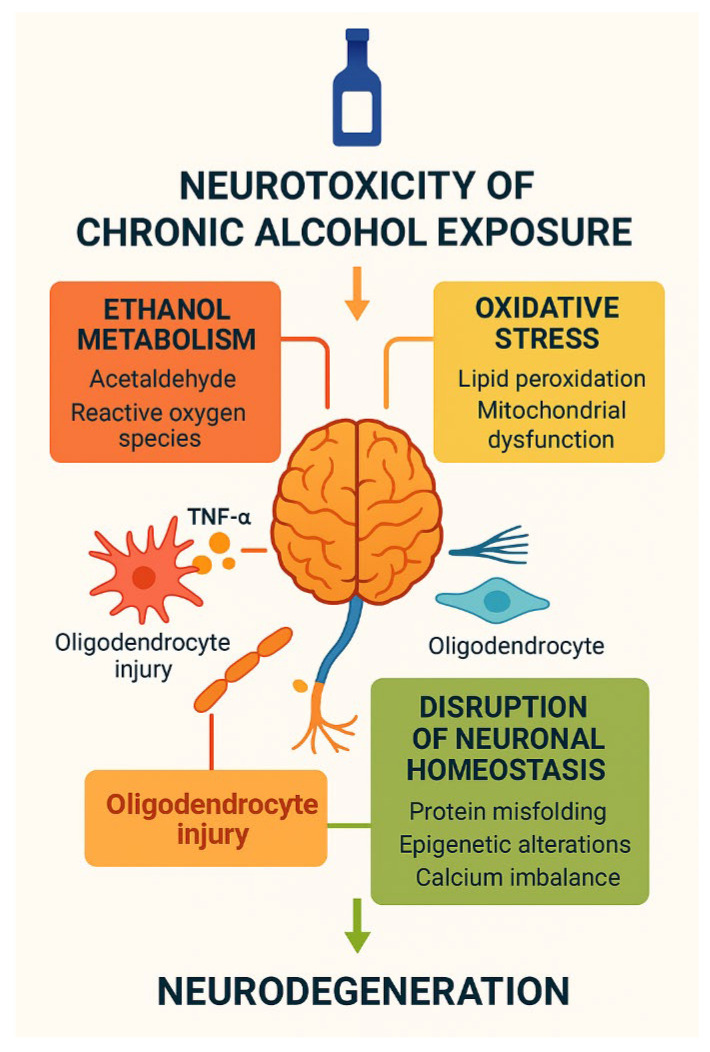
Neurotoxicity of chronic alcohol exposure. Schematic overview of the neurotoxic effects of chronic alcohol exposure. Ethanol metabolism generates acetaldehyde and reactive oxygen species, inducing oxidative stress, lipid peroxidation, mitochondrial dysfunction, oligodendrocyte injury, and calcium imbalance. These processes disrupt neuronal homeostasis, promote protein misfolding and epigenetic alterations, and ultimately converge on progressive neurodegeneration. Created by the authors in Adobe Illustrator version 27.5, 2023 (created on 27 October 2025).

In conclusion, ethanol-induced neurotoxicity remains a preventable yet under-recognised contributor to the global neurodegenerative burden. Continued efforts to elucidate its underlying molecular mechanisms and to translate these findings into evidence-based clinical applications are essential for mitigating the profound impact of alcohol-related brain disorders (Figure 5).

## Figures and Tables

**Table 1:** Major mechanisms of alcohol-induced neurotoxicity. Summary of core cellular and molecular pathways described in Section 2, Section 3, Section 4, Section 5, Section 6, Section 7, Section 8 and Section 9 through which ethanol disrupts brain structure and function.

Mechanism	Molecular Pathways	Consequences
Oxidative stress	Excess ROS/RNS production, NOX activation, lipid peroxidation, impaired antioxidant defence	DNA and mitochondrial DNA damage, loss of membrane integrity, synaptic loss, impaired neurotransmission
Mitochondrial dysfunction	ETC impairment, reduced ATP synthesis, mPTP opening, altered Ca^2+^ buffering, Drp1-mediated fission, defective mitophagy	Bioenergetic failure, cytochrome c release, activation of intrinsic apoptosis, axonal transport and synaptic transmission deficits
ER stress and protein misfolding	PERK–eIF2α–ATF4 pathway activation, CHOP induction, unfolded protein response (UPR) dysregulation, impaired UPS/autophagy	Accumulation of misfolded/aggregated proteins, ER-stress-induced apoptosis, loss of proteostasis in neurons and glia
Calcium dysregulation	NMDA/AMPA overactivation, opening of voltage-gated Ca^2+^ channels (VGCCs), ER Ca^2+^ release, mitochondrial Ca^2+^ overload	Excitotoxic neuronal injury, cytoskeletal disintegration, mPTP opening, amplification of ROS production
Neuroinflammation	TLR4–MyD88/TRIF–NF-κB activation, microglial cytokines (TNF-α, IL-1β, IL-6), MCP-1/CCR2 chemokine signalling, recruitment of peripheral immune cells	Synaptic pruning and loss, gliosis, disruption of neuron–glial communication, chronic neuronal loss
Epigenetic remodeling	DNMT/HDAC imbalance, altered DNA methylation, histone acetylation/methylation changes, dysregulated miRNA profiles	Stable reprogramming of gene expression, impaired neurotrophic and survival signalling, increased susceptibility to stress and neurodegeneration
White matter injury and oligodendrocyte vulnerability	Ethanol-induced oxidative stress, mitochondrial dysfunction and lipid peroxidation in oligodendrocytes; impaired myelin protein expression; disrupted OPC proliferation and differentiation; microglial cytokines (TNF-α, IL-1β, IL-6); astrocytic connexin dysfunction; activation of Wnt/β-catenin and Notch signalling	Myelin thinning and loss, reduced remyelination capacity, axonal conduction deficits, disconnection of cortico–subcortical circuits, cognitive slowing and processing-speed deficits
Apoptotic signalling	Bax translocation, mitochondrial outer membrane permeabilization (MOMP), caspase-9/3 activation, TRAIL/death-receptor pathway activation, suppression of PI3K/Akt/mTOR survival signalling	Programmed neuronal cell death, synaptic and circuit degeneration

Abbreviations: ROS—reactive oxygen species; RNS—reactive nitrogen species; NOX—NADPH oxidase; ETC—electron transport chain; mPTP—mitochondrial permeability transition pore; Ca^2+^—calcium ion; ER—endoplasmic reticulum; UPR—unfolded protein response; UPS—ubiquitin–proteasome system; TNF-α—tumour necrosis factor alpha; IL-1β—interleukin-1 beta; IL-6—interleukin-6; TLR4—Toll-like receptor 4; MCP-1—monocyte chemoattractant protein-1; CCR2—C-C chemokine receptor type 2; DNMT—DNA methyltransferase; HDAC—histone deacetylase; miRNA—microRNA; PI3K—phosphoinositide 3-kinase; Akt—protein kinase B; mTOR—mechanistic target of rapamycin.

**Table 2:** Neurotransmitter system dysregulation in alcohol exposure. Summary of predominant ethanol-induced alterations in major neurotransmitter systems and their functional consequences, with emphasis on chronic and heavy drinking patterns.

Neurotransmitter System	Ethanol-Related Changes (Dose/Pattern)	Functional and Behavioural Consequences
GABAergic	Acute low–moderate doses: positive allosteric modulation of GABA_A receptors, enhanced inhibitory tone. Chronic and heavy use: downregulation and altered subunit composition of GABA_A receptors, reduced inhibitory transmission, impaired GABA release.	Sedation and anxiolysis in acute use; during chronic exposure and withdrawal neuronal hyperexcitability, anxiety, seizures, heightened vulnerability to excitotoxic injury.
Glutamatergic	Acute exposure: transient inhibition of NMDA receptor function. Chronic and binge drinking: upregulation and hyperactivation of NMDA receptors, elevated extracellular glutamate, impaired glutamate uptake by astrocytes.	Excitotoxicity, Ca^2+^ overload, mitochondrial dysfunction, dendritic spine loss, cognitive impairment and increased risk of neurodegeneration
Dopaminergic (mesolimbic)	Acute exposure: increased phasic dopamine release in ventral tegmental area (VTA) → nucleus accumbens (NAc). Chronic use: blunted dopaminergic responses, altered firing patterns, accumulation of ethanol-derived metabolites (e.g., salsolinol).	Reward dysregulation, reinforcement of alcohol-seeking, anhedonia and motivational deficits during withdrawal, dependence-related synaptic plasticity
Serotonergic	Dose and pattern-dependent alterations in 5-HT release and turnover; changes in 5-HT_1A/5-HT_2A receptor expression and signalling in prefrontal and limbic regions, particularly with chronic exposure.	Dysregulation of mood, anxiety, impulsivity and sleep–wake cycles; contribution to comorbid depression and anxiety in AUD.
Noradrenergic	Disruption of locus coeruleus noradrenergic output, especially during chronic use and withdrawal; increased noradrenergic tone and receptor sensitivity in stress-related circuits	Autonomic hyperarousal, irritability, insomnia and stress-related relapse; amplification of withdrawal symptoms and negative affect.
Cholinergic interneurons (striatum/NAc)	Modulation of ethanol-sensitive GABA_A receptors on striatal and nucleus accumbens cholinergic interneurons; altered acetylcholine release and integration of cortical and dopaminergic inputs	Abnormal gating of striatal circuits, disturbed action selection and habit formation, reinforcement of alcohol-seeking and long-term synaptic instability

Abbreviations: GABA—γ-aminobutyric acid; GABA_A—GABA type A receptor; NMDA—N-methyl-D-aspartate receptor; VTA—ventral tegmental area; NAc—nucleus accumbens; 5-HT—serotonin; AUD—alcohol use disorder; Ca^2+^—calcium ion.

**Table 3:** Sex-specific vulnerabilities and the main contributing biological factors are summarized in Table 3. Overview of biological sex differences determining susceptibility to alcohol-related brain injury.

Sex/Comparison	Biological Determinants	Neurotoxic Outcomes
Female	Higher blood alcohol concentration (BAC) for a given dose due to lower gastric ADH activity and higher body fat; estrogen-enhanced TLR4–NFκB-driven inflammation; greater oxidative stress and microglial activation; enhanced mitochondrial dysfunction and Ca^2+^ dysregulation under equivalent exposure; stronger microglial and astrocytic inflammatory responses.	Greater white matter and hippocampal damage; earlier onset of cognitive decline; increased anxiety- and depression-like symptoms at lower cumulative exposure; increased oligodendrocyte and myelin damage, reduced remyelination capacity, and greater vulnerability to disconnection/processing-speed deficits.
Male	Greater absolute alcohol intake and more frequent binge patterns; higher gastric ADH activity and total body water; testosterone-related support of PI3K/Akt signalling and antioxidant capacity; lower basal cytokine reactivity	Higher total lifetime dose before overt deficits; relatively slower structural decline, but pronounced executive and decision making impairments with prolonged heavy use
Female > Male	Enhanced mitochondrial dysfunction and Ca^2+^ dysregulation in several brain regions under equivalent exposure; stronger microglial and astrocytic inflammatory responses	Increased oligodendrocyte and myelin damage; reduced remyelination capacity; greater vulnerability to disconnection and processing-speed deficits
Male ≈ Female	Shared epigenetic remodeling (DNMT/HDAC imbalance, altered DNA methylation and histone marks, dysregulated miRNA profiles); overlapping excitotoxic, oxidative and apoptotic cascades	Convergent long-term transcriptional reprogramming; persistent neuroinflammation and synaptic loss in both sexes

Abbreviations: ADH—alcohol dehydrogenase; BAC—blood alcohol concentration; TLR4—Toll-like receptor 4; NFκB—nuclear factor kappa B; PI3K—phosphoinositide 3-kinase; Akt—protein kinase B; DNMT—DNA methyltransferase; HDAC—histone deacetylase; miRNA—microRNA; Ca^2+^—calcium ion.

**Table 4:** Therapeutic strategies targeting alcohol-induced neurotoxicity. Overview of interventions and their molecular targets, illustrating translational potential.

Therapeutic Strategy	Molecular Target	Rationale/Intended Effect
Antioxidants (N-acetylcysteine, glutathione donors)	ROS, lipid peroxidation	Reduce oxidative damage and lipid peroxidation, protect mitochondria
Mitochondrial stabilizers (coenzyme Q10, SS-peptides, MitoQ)	ETC complexes, mitochondrial membrane potential (ΔΨm)	Restore mitochondrial efficiency, maintain ΔΨm, prevent cytochrome c release and apoptosis
HDAC inhibitors/DNMT modulators	Epigenetic markers (histones, DNA methylation)	Normalize gene expression, reverse maladaptive epigenetic remodeling
TLR4 antagonists/microglial inhibitors	Microglial NFκB pathway	Reduce neuroinflammation and cytokine release, limit microglial activation
Calcium channel blockers	NMDA and VGCC-mediated Ca^2+^ influx	Prevent excitotoxicity and Ca^2+^-dependent mitochondrial injury
Memantine/other NMDA antagonists	NMDA receptor signaling	Attenuate glutamate-driven excitotoxic injury
BDNF enhancers/TrkB agonists, PI3K/Akt/mTOR activators	Neurotrophic and survival signaling	Support neuronal survival and plasticity, promote structural and functional recovery

Abbreviations: ROS—reactive oxygen species; ETC—electron transport chain; ΔΨm—mitochondrial membrane potential; HDAC—histone deacetylase; DNMT—DNA methyltransferase; TLR4—Toll-like receptor 4; NFκB—nuclear factor kappa B; NMDA—N-methyl-D-aspartate receptor; VGCC—voltage-gated calcium channel; BDNF—brain-derived neurotrophic factor; TrkB—tropomyosin receptor kinase B; PI3K—phosphoinositide 3-kinase; Akt—protein kinase B; mTOR—mechanistic target of rapamycin; Ca^2+^—calcium ion.

## Data Availability

No new data were created or analyzed in this study. Data sharing is not applicable to this article.
